# Testing geometry and 3D perception in children following vision restoring cataract-removal surgery

**DOI:** 10.3389/fnins.2022.962817

**Published:** 2023-01-11

**Authors:** Amber Maimon, Ophir Netzer, Benedetta Heimler, Amir Amedi

**Affiliations:** ^1^The Baruch Ivcher Institute for Brain, Cognition, and Technology, Baruch Ivcher School of Psychology, Reichman University, Herzliya, Israel; ^2^The Ruth & Meir Rosenthal Brain Imaging Center, Reichman University, Herzliya, Israel; ^3^Gonda Brain Research Center, Bar-Ilan University, Ramat Gan, Israel; ^4^Center of Advanced Technologies in Rehabilitation (CATR), Sheba Medical Center, Ramat Gan, Israel

**Keywords:** vision restoration, sensory perception, sensory development, visual perception, cataract removal, visual development, geometry, 3D perception

## Abstract

As neuroscience and rehabilitative techniques advance, age-old questions concerning the visual experience of those who gain sight after blindness, once thought to be philosophical alone, take center stage and become the target for scientific inquiries. In this study, we employ a battery of visual perception tasks to study the unique experience of a small group of children who have undergone vision-restoring cataract removal surgery as part of the Himalayan Cataract Project. We tested their abilities to perceive in three dimensions (3D) using a binocular rivalry task and the Brock string task, perceive visual illusions, use cross-modal mappings between touch and vision, and spatially group based on geometric cues. Some of the children in this study gained a sense of sight for the first time in their lives, having been born with bilateral congenital cataracts, while others suffered late-onset blindness in one eye alone. This study simultaneously supports yet raises further questions concerning Hubel and Wiesel’s critical periods theory and provides additional insight into Molyneux’s problem, the ability to correlate vision with touch quickly. We suggest that our findings present a relatively unexplored intermediate stage of 3D vision development. Importantly, we spotlight some essential geometrical perception visual abilities that strengthen the idea that spontaneous geometry intuitions arise independently from visual experience (and education), thus replicating and extending previous studies. We incorporate a new model, not previously explored, of testing children with congenital cataract removal surgeries who perform the task *via* vision. In contrast, previous work has explored these abilities in the congenitally blind *via* touch. Taken together, our findings provide insight into the development of what is commonly known as the visual system in the visually deprived and highlight the need to further empirically explore an amodal, task-based interpretation of specializations in the development and structure of the brain. Moreover, we propose a novel objective method, based on a simple binocular rivalry task and the Brock string task, for determining congenital (early) vs. late blindness where medical history and records are partial or lacking (e.g., as is often the case in cataract removal cases).

## 1. Introduction

“You’ll learn,” the blind man answered. “There is much to learn in the world.” And indeed, as discovered by the protagonist in Wells, 1921 short story “The Country of the Blind,” we have much to learn from the blind and the visually impaired. Particularly with regard to the neuroscience of vision and the development of the brain and the senses. Today, actual attempts at restoring vision allow for true exploration concerning these themes. In particular, by way of cataract removal. Though cataract removal methods and techniques have been documented for hundreds of years–with one of the first reported cases taking place as early as 1615 (Leffler et al., 2021), case studies and reports on the visual abilities and experiences of people who undergo cataract removal surgeries are still relatively few and far between (Fine et al., 2003; Ostrovsky et al., 2006).

Cataracts are the leading cause of vision impairment in children, particularly those residing in low-income countries worldwide (World Health Organization [WHO], 2021). Several humanitarian efforts are currently underway to change this unfortunate circumstance and rectify the situation. Among these projects is project Prakash (Thomas, 2011; Sinha, 2013; Sinha et al., 2013), a project with humanitarian and scientific goals led by Prof. Pawan Sinha, and the Himalayan Cataract Project (Welling et al., 2013; Brant et al., 2021), founded by Drs. Geoffrey Tabin and Sanduk Ruit that aims to eradicate curable blindness.

David Hubel and Torsten Wiesel, who later won the 1981 Nobel Prize for this work, found that deprivation of visual input in the first few months of the lives of animals (such as cats and monkeys) led to irreversibly abnormal visual processing (Wiesel and Hubel, 1965; Hubel et al., 1977; LeVay et al., 1980). They found that when monocularly deprived of vision, the percentage of cells driven by the sensory-deprived eye is reduced (Wiesel and Hubel, 1963). When binocularly deprived of vision, they found a decrease in the number of binocularly influenced cells. They suggested that this indicates “a deterioration of innate connections subserving binocular convergence” (Wiesel and Hubel, 1974, p. 1060).

Following from these findings, Hubel and Wiesel (1963) concluded that while there is a basic organization in place at birth, for proper development and visual processing, visual input is necessary. They thus formulated the critical periods hypothesis, which postulates that there is a critical period for developing the sense of vision (and other senses). If sensory information is deprived during the critical period, the neuronal morphology and connectivity are altered in such a way that the sense cannot be gained or recovered at a later stage (Wiesel and Hubel, 1965; Hubel and Wiesel, 1970). In humans, while the greatest chance of visual recovery in the case of detected and treated visual abnormality is under the age of 5 (Siu and Murphy, 2018), the critical period for binocularity was thought to decrease by age 6–8 (Aslin and Banks, 1978), with some studies pointing to the end of the critical period for stereopsis as falling between the age of 4–5 (Fawcett et al., 2005). Despite this, research conducted specifically on congenital cataract removal by Prof. Pawan Sinha and others indicates that the human brain “retains the capacity” for the acquisition of vision even after extended sensory deprivation during critical periods (Held et al., 2011). A wealth of research indicates that neuroplasticity can bring about enhanced development in the intact skills and abilities of the sensory deprived (Amedi et al., 2005; Heimler et al., 2014; Heimler and Amedi, 2020). Further support for this comes from studies showing compensatory neuroplasticity, for example, switching of tasks performed by a specific brain area leading to enhancement in high-level cognitive functions, such as memory or language (Amedi et al., 2003; Bedny et al., 2011 or memory in a causal relationship Amedi et al., 2004), or neuroplasticity that underlies the ability to perform substitution of one sense by another. Contemporary research on blind users trained with sensory substitution devices that translate vision to audition show activation in category-specific visual areas when using the devices for various tasks, such as identification of objects (Striem-Amit et al., 2012a), letters (Reich et al., 2011), and numbers (Abboud et al., 2015).

A cataract is a lens opacity that causes visual impairment, sometimes to complete blindness (Grałek et al., 2007). Cases of visual restoration following cataract removal represent the true core of both the philosophical and scientific debate on sight, the senses, and neuroplasticity. Would one who underwent surgery that allowed them to gain a previously inexperienced sense of vision be able to “know” what they were seeing? If so, how rapidly and to what level would the ability to use this knowledge, for example, for perceiving three dimensions (3D) vision and geometry, come about? These findings are also interesting for the nature vs. nurture debate concerning visual properties. This debate dates back to the time of John Locke and his acquaintance William Molineux, who pondered in correspondence whether a blind person who could recognize objects by touch would be able to recognize those same objects by vision, were their vision miraculously restored (Locke, 1847; Ferretti and Glenney(eds), 2021). We aim to weigh in on several core questions in this case study. Would children blind from birth in one or both eyes gain true visual properties? If so, to what extent and how similar or different is their visual experience from those of the normally sighted? Would they achieve the level of visual knowledge experienced by the normally sighted children?

In addition, we specifically explore some still-open questions at the forefront of research conducted with vision restoration patients. Would these children have 3D vision? Fine et al. (2003) conducted a case study that showed that long-term visual deprivation leads to deficits in processing complex forms, specifically 3D. Would the children be able to correspond what they now see with what they feel through touch? Prior research indicates, for example, that the cross-modal transfer of information between the tactile sense and the newly acquired visual one does not develop immediately. However, it develops within a few days (Held et al., 2011). Later research indicates that this correspondence occurs quicker than previously thought (Chen et al., 2016). Would these children be susceptible to visual illusions? It was commonly believed that susceptibility to visual illusions is visual experience-dependent (Gillam, 1980). Yet, a study showed that children who underwent cataract removal surgery (as part of project Prakash) are susceptible to certain illusions immediately after surgery (Gandhi et al., 2015). Moreover, how would they perform on tasks requiring the spatial grouping of visual geometric cues? Research conducted with haptic geometric cues has led to conflicting conclusion. On the one hand, Marlair et al. (2021) showed lower performance in the blind than the sighted, but on the other, Heimler et al. (2021) showed similar performance in the blind as in the sighted.

This paper aims to provide insight into these key questions, to some extent, through the individual experiences of eight children who underwent cataract-removal surgery in Quiha hospital in Ethiopia as part of the Himalayan Cataract Project. We were able to explore the children’s visual state a few days after surgery (but due to the circumstances–not before) to shed some light on the relationship between the behavioral and the neurological. A case study is particularly warranted in these circumstances due to the exceptional nature of these cases. The extensive battery of tests we employ allows for ascertaining the fine details of the children’s visual experience. We believe this paper serves as a springboard for more research in this challenging field and paves the way for a deeper understanding of the development of vision and the senses in general.

In addition, we wish to propose a novel and more objective method for determining congenital (early) vs. late blindness in children undergoing cataract-removal surgery. In these cases, particularly in, but not limited to, low and middle-income countries, it is often difficult to determine the precise medical background of the children, and their clinical state is often not adequately documented, leaving the medical and rehabilitative staff often dependent on reports of the parents alone. We suggest utilizing the findings of this study, particularly concerning the binocular rivalry and the Brock String task, as a method for distinguishing cases of congenital (early) and late blindness in children following cataract surgery.

## 2. Materials and methods

### 2.1. Participants and ethics

Eight children participated in the study, all of whom underwent vision restoring or rehabilitating ophthalmological surgery in the days before the study as part of the Himalayan Cataract Project (see Table 1 for more details). For the purpose of this study, childhood is defined as below the universally accepted age of 18 (McGoldrick, 1991). The children presented with various visual impairments but had no other known diseases or medical conditions. RS (male, 11 years old) and HB (male, 13 years old) had congenital cataracts in both eyes. IG (male, 14 years old) had a congenital cataract in one eye. OB (male, 7 years old), GA (male, 7 years old), AC (female, 10 years old), AB (male, 12 years old), and GH (female, 10 years old) had trauma-induced cataracts in one eye. While RS and HB had been blind from birth in both eyes, and IG blind from birth in one eye, the others had much shorter periods of vision loss, between 2 weeks to a month, before surgery (see Table 1 for demographic and medical information about the children). All the children underwent the operation 4–6 days before the study. The children’s legal guardians gave informed consent to their participation in this study. In addition, the study was conducted within the hospital setting while the children were under the care of the hospital staff and adhered to the ethical guidelines of the declaration of Helsinki.

**TABLE 1:** Demographic and medical information about the children.

Subject	Gender	Age	Days from surgery	Schooling	Type of cataract	Reported visual acuity
RS	Male	11	5	None	Congenital	*Pre-op*: Right: Hand motion (HM) Left: HM *Post-op*: Right: 6/36 Left: 6/6
HB	Male	13	4	2 years	Congenital	*Pre-op*: Right: 1/2 M Left: HM *Post-op*: Right: 6/18 Left: 6/12
IG	Male	14	6	2 years	Congenital/ one eye	*Post-op*: Right: Counting fingers (CF) 1M Left: 6/6
OB	Male	7	6	None	Acquired/ one eye	*Pre-op*: Right: Light perception (LP) *Post-op*: Right: 6/6
GA	Male	7	5	None	Acquired/ one eye	*Pre-op*: Left: LP *Post-op*: Left: 6/12
AC	Female	10	5	2 years	Acquired/ one eye	*Post-op*: Left: CF 2M Lacking color perception
AB	Male	12	5	1 year	Acquired/ one eye	*Post-op*: Right: CF 2M
GH	Female	10	5	2 years	Acquired/ one eye	*Post-op*: Right: 6/6 Left: 6/6

### 2.2. Binocular rivalry

**FIGURE 1:**
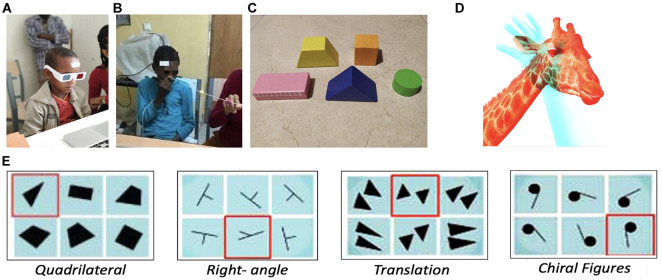
**(A)** A child in the study that had cataract removal surgery 4–6 days prior to undergoing the binocular rivalry task. **(B)** A child in the study undergoing the Brock string task and binocular rivalry task. **(C)** Geometrical three dimensional (3D) shapes used for the three-dimensional cross-modal object recognition task. **(D)** Superimposed images were used to test binocular rivalry. **(E)** Spatial grouping task based on geometric cues from Dehaene et al. (2006) (detection of the outlier in each group of geometric cues, for example, the triangle among the quadrilaterals).

All subjects performed a binocular rivalry task and a depth perception task. The subjects wore classic (generic) paper 3D viewing glasses in the binocular rivalry task. They were presented with stimuli consisting of two superimposed cartoon figures (cartoon figures were used as stimuli as the subjects were children) in red and blue (see examples in Figure 1D). We asked participants to close one eye at a time to see each figure separately and then to look at the image on the screen with both eyes and report whether they see the two figures alternating. In those with normally developed vision, the simultaneous presentation of two different images in two colors superimposed through the red/blue filter leads to a well-documented perceptual phenomenon of image dominance switching (Wade, 1998). The perceptual dominance of the images changes such that only one image is perceived at a time, with the images switching between them (coming in and out of active perception) every few seconds (Miller et al., 2000; Pettigrew, 2001; Blake and Logothetis, 2002).

On the other hand, when one eye or the other is covered, only one image is perceived at a time without changing. This phenomenon is closely correlated with the two-dimensional information presented to our eyes from the outside world, which is combined into a single three-dimensional representation in the brain (Levelt, 1965). In this study, the children were instructed to look at the images first with one eye, then with the other, then with both eyes while fixating their gaze on the fixation cross at the top center of the image (Figure 1A).

### 2.3. Depth perception with the Brock string task

In addition to the binocular rivalry task, all children performed a Brock string task to test their ability to converge the information acquired by their two eyes to create binocular 3D vision (Brock, 1955). The instrument employed in the task is a white string with three beads, one green, one yellow, and one red, placed along the string’s length at different intervals. The string and beads used for the task were homemade and not commercial instruments. One end of the string is held precisely at the tip of the subject’s nose, while the other is placed at a fixed location with the string pulled tautly. In this task, the experimenter points sequentially at the three balls, and the participant must gaze at them, reporting what they (see Figure 1B). Participants prepared for ∼1 min using the string: the experimenter pointed sequentially to the different balls on the thread, and the participants needed to direct their gaze to the ball pointed to by the experimenter. If the beads appear double to the subject, then it indicates an inadequacy in the convergence of visual input. If one has binocular depth perception, s/he will start to see two lines rather than only one line.

### 2.4. Spatial grouping based on geometric cues

Four children, RS, HB (the two who had bilateral congenital cataracts removed), AC and GH (who had trauma-induced cataracts removed and were close in age to RS and HB), performed a spatial grouping task based on geometric cues (Dehaene et al., 2006) in which they are shown six images–five images depicting a specific geometric concept, and one outlier which does not abide by the given regularity (for example, right angles, or parallel lines). The children were asked to identify the outlier among the given geometric groups (see, for instance, Figure 1E).

### 2.5. Cross-modal object recognition

RS, HB, and IG were also tested for cross-modal object recognition. During this task, they were asked to feel a 3D geometrically shaped wooden shape (store-bought generic wooden blocks) they had never been exposed to before (using touch alone–without seeing the shape as it was placed in a black, opaque bag) corresponding to some of the shapes in a geometrical cues task (see below). They were asked to look at one shape and report whether it was the same or different from the shape they were touching; then, to match among alternatives: look at two shapes and point to the one that matched the tactile shape they were touching (Figure 1C–the same wooden shapes they touched were placed on a table in front of them). This task was repeated twice: once using 3D real shapes for visual matching; once using 2D figures of the same shapes presented on the computer.

### 2.6. Visual illusions

**FIGURE 2:**
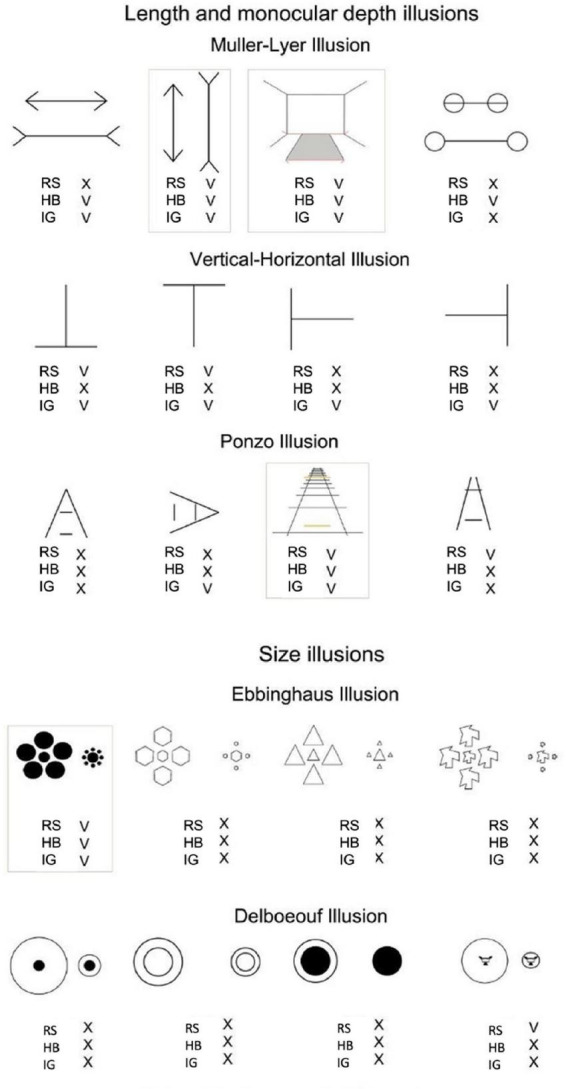
The single subject susceptibility to visual illusions. V represents the subject’s susceptibility to the illusion, and X means that the subject was not susceptible to the illusion.

These three children were also tested on their perception of visual illusions. The children were presented with classic visual illusions: Length illusions: the Muller-Lyer (1889) illusion, the vertical-horizontal illusion (Künnapas, 1955), and the Ponzo (1911), Size illusions: Delboeuf (1865) and Ebbinghaus (1902) illusions. In the length and depth illusions, the children were asked whether one of the two lines appeared to be longer, while in size illusions, they were asked whether one of the two circles looked bigger (see Figure 2).

## 3. Results

### 3.1. Binocular rivalry

The two children with bilateral congenital cataracts removed (RS and HB) did not show binocular rivalry despite reporting that they accurately saw each image with the two eyes separately, meaning that they did not see the two images alternating at any point of the task. IG, who had a congenital cataract in one eye removed, did not report binocular rivalry. AC had a unilateral trauma-induced cataract removed, lacked color perception in the eye, and did not show binocular rivalry. The other children showed binocular rivalry when tested 4–6 days after surgery.

### 3.2. Depth perception with the Brock string task

If one has binocular depth perception, they will see two lines crossing instead of only one line after some time. The two children with bilateral congenital cataracts removed (RS and HB) had no binocular depth perception. Four of the five children with unilateral trauma-induced cataract removals did have depth perception. IG, who had a congenital cataract in one eye removed, did not have depth perception during the task.

### 3.3. Spatial grouping based on geometric cues

**FIGURE 3:**
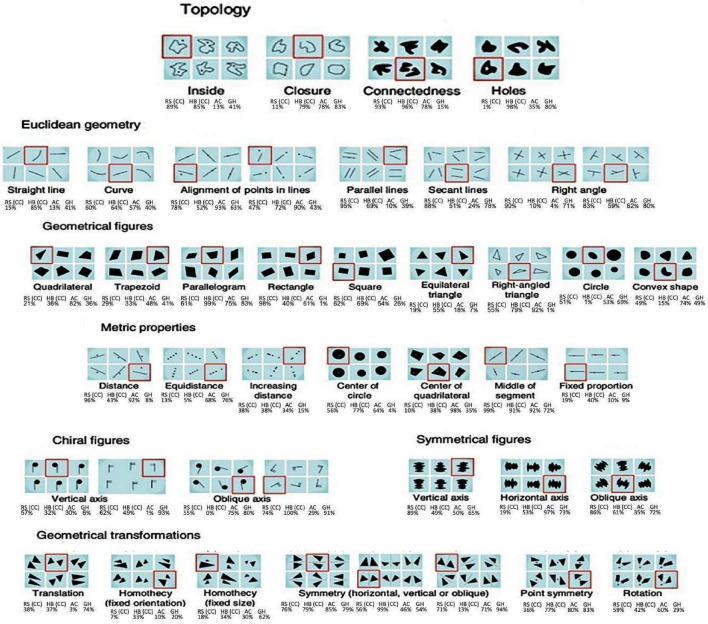
The single subject average results on the spatial grouping task based on geometric cues from Dehaene et al. (2006) (chance level is 16.6% in these tasks).

**FIGURE 4:**
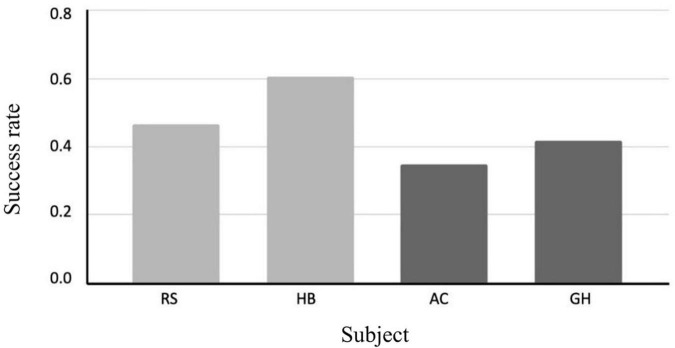
The single subject grand average success on the spatial grouping task.

RS and HB (who had bilateral congenital cataracts removed), compared to AC and GH, who had unilateral trauma-induced cataracts removed, performed this task. Correct identification of the outlier was considered to be a “success.” All the children tested had no or minimal prior schooling. The results showed that RS and HB (congenital cataracts) outperformed AC and GH on this task with an average success of 53% vs. 38%, much higher than the chance level of 16.6%. Of course, it is difficult to reach generalized conclusion with such a low number of subjects and trials, given the nature of this field research (Figures 3, 4).

### 3.4. Cross-modal object recognition

RS and HB (who had bilateral congenital cataracts removed), and IG (who had a unilateral congenital cataract removed), underwent testing for cross-modal object recognition. If the child correctly pointed to the visual shape that matched the tactile shape they were touching, it was considered a “success.” When tested 4–6 days after surgery, the children after bilateral cataract removals showed very high accuracy in both the 2D and the 3D conditions. RS and HB each succeeded in 9/10 trials with an accuracy of 90% (much higher than the 50% chance level), and IG succeeded in 6/10 with an accuracy of 60%.

### 3.5. Visual illusions

A total of 4–6 days after surgery, RS, HB, and IG were tested on visual illusions. RS and HB (who had bilateral congenital cataracts removed) showed higher susceptibility to length illusions (Muller-Lyer, Vertical-Horizontal, and Ponzo) than to size illusions (Ebbinghaus, Delboeouf). This test was binary. Either the child perceived the illusion or not. Higher susceptibility, in this case, refers to the fact that the children were influenced more by the length illusions than the size illusions (as seen in Figure 2).

## 4. Discussion

In this case study, eight children underwent a battery of numerous visual tests and tasks in a challenging field setting, including the classic binocular rivalry red/blue filtered glasses task and the Brock string task of depth perception. Of the eight children who participated in the study, Two of them were born with bilateral congenital cataracts, thereby experiencing true unobscured sight for the first time in their lives only in the few days preceding the study. One child was unilaterally congenitally blind, thereby experiencing binocular vision for the first time in his life. The remaining five were normally sighted children who lost vision in one eye due to trauma-induced cataracts. As such, these children served as important control cases, representing age-matched children with normal visual development during the standard critical periods. The children with congenital cataracts were the only ones in the group not to display either binocular rivalry or depth perception on the Brock string task. In contrast, the other cases of trauma-induced and later onset/short-term cataracts did show these abilities. Out of the group, the two children with congenital cataract removals (bilateral and unilateral) were also tested on visual illusions and cross-modal correspondence. The children were susceptible to some depth illusions relying on monocular cues, such as the Ponzo illusion. They showed high accuracy in both the 2D and the 3D conditions of the cross-modal correspondences task (the two with bilateral congenital cataracts showed nearly ceiling-level accuracy). In addition, the two children with bilateral cataract removals were compared to two children with trauma-induced cataract removals in a task of spontaneous use of geometric cues. In this task, the children with bilateral congenital cataract removals displayed an even higher success rate than their peers.

### 4.1. The results in the context of the theory of critical periods

Hubel and Wiesel’s Nobel prize-winning studies claim that sensory deprivation, specifically of visual input in the early stages of life, would prevent the rehabilitation of vision later in life (Wiesel and Hubel, 1965; Hubel et al., 1977; LeVay et al., 1980). On the one hand, the visual properties we observed in the children who were deprived of vision during the critical period (those who underwent bilateral or unilateral congenital cataract removals) support the theory of critical periods (with regard to binocular vision and depth perception in particular). But on the other hand, some findings we observed in other tasks hint at a different interpretation overall. It has been suggested that the unnatural, immediate increase in visual acuity following congenital cataract removal does not follow the course of events of vision acquisition in newborns, which in itself may delay the proper encoding of visual information in the period directly following the surgery (Vogelsang et al., 2018).

In this study, when tested a few days after the surgery, the children who underwent congenital cataract removal showed neither binocular rivalry nor depth perception on the Brock string test. The obvious and clear implication of this is that, as expressed by Bach-y-Rita (1972), we see with our brains, not with our eyes. Though the children’s eyes were no longer occluded, and they achieved moderate-mild visual acuity (the WHO defines mild visual acuity as worse than 6/12 to 6/18 and moderate visual acuity as worse than 6/18 to 6/60), their higher-level visual processing of the information was not fully established.

Interestingly, on the visual illusion tasks in our study, the children who had congenital cataracts removed were susceptible to some of the depth illusions that rely on monocular depth cues, such as the Ponzo illusion. This might indicate that at the time of testing, a few days after surgery, the children were at an intermediate stage of visual recovery. This is further strengthened by our findings concerning cross-modal object recognition and grouping based on geometric cues, and by animal research that indicates sensory-motor stimulation can promote recovery from visual deprivation (Baroncelli et al., 2010; Levelt and Hübener, 2012). The children’s success on these tasks could represent the initial stages of development of a sense of 3D in the visual domain. It is possible that recovery of bilateral depth is not as quick as other aspects of visual recovery, compared to the results of the other tasks, which the children were able to perform at a level similar to the children who underwent trauma-induced cataract removal. This point is raised with caution, as we lack information concerning the continued development of the children’s vision at a later time. Yet we believe this indicates that further research into the long-term visual recovery of children who have undergone bilateral congenital cataract removal is particularly warranted.

### 4.2. A novel, objective method for determining congenital (early) vs. late blindness

As described above, the children’s results on the binocular rivalry task and the Brock string task are particularly interesting. Taken together, these two tasks seem to be the primary distinguishing factor between congenitally blind children and those who developed cataracts later in life. We propose utilizing these two simple, straightforward tests as a method of making this differentiation precisely in the field. This is particularly important for, but not limited to, projects operating in low or middle-income countries where access to medical records and documentation is less readily available or even scarce (Röder et al., 2021).

To promote and reliably conduct research involving people who have undergone cataract removal or other surgeries and procedures for sight restoration in childhood or even adulthood. Conducting research with these individuals necessitates a very high degree of certainty that the study participants were indeed devoid of vision from birth/very early life, specifically during the critical periods (Röder et al., 2021).

Following our study’s findings, we propose a novel method for retroactively identifying individuals born congenitally blind. Potential subjects can be screened on the binocular rivalry task and the Brock string task in combination a few days after surgery. Our findings would allow the researcher to confirm or disaffirm a congenital cataract diagnosis retroactively since children with trauma-induced cataracts later in childhood could perform these two specific tasks while children with congenital cataracts were not.

### 4.3. Replication and extension of previous studies on cross-modal correspondence following congenital cataracts

The children’s results on the cross-modal object recognition tasks and the spatial grouping based on geometric cues have significant scientific and philosophical implications. Cross-modal object recognition tasks are historically based on a philosophical thought experiment known as Molyneux’s problem (Ferretti and Glenney(eds), 2021). Molyneux, whose wife was blind, pondered upon whether a blind person who could recognize objects by touch would be able to recognize those same objects by vision, were his/her vision miraculously restored. Molyneux pondered whether sighted and touch can or cannot be linked immediately upon first sight Molyneux’s answer to his proposed question was that they cannot, a stance backed by his friend Locke (1847) and further agreed upon and expanded by George Berkley. Berkley (1709) further stated that visual experience gained by the blind upon visual restoration represents a “new set of ideas, perfectly distinct and different from the former, and which can in no sort make themselves perceived by touch.” This philosophical stance represents that of the empiricists, who opposed the idea that there are innate amodal mechanisms in common between the senses and that true sensory knowledge can only be gained through modality-specific sensory experience.

Previous research conducted on children following congenital cataract removal surgeries through Project Prakash found evidence that was consistent with Molyneux’s idea in that the children could not immediately correspond between what they saw and what they had felt (Held et al., 2011). Yet they showed that the children’s abilities to perform this matching improved rapidly, developing within a few days. Another study by Chen et al. (2016) also showed very rapid development of these abilities in a child who had undergone cataract-removal surgery in under 2 days, concluding that the merging between the senses is “prearranged.” Our results are consistent with these findings, as the children in our study reached nearly top performance on this task when tested a few days following their surgeries after having never encountered these items in the visual domain. In addition, unlike Held et al. (2011), the stimuli used in our study were naturally occurring geometric shapes, further correlated to the task of spatial grouping by geometric cues (as expanded upon below). Held et al. (2011) suggested that the performance improvement may be due to their ability to create a three-dimensional visual representation. Yet, the children in our study (who had congenital cataracts removed) could not create three-dimensional representations at the stage at which they could perform with very high accuracy on the cross-modal object recognition task.

So while our findings are consistent with those of Held et al. (2011) who show the development of this ability in such a consistently rapid way. We interpret these findings differently with respect to the conclusion drawn with respect to Molyneux’s problem. We claim that the extremely rapid development of this ability, within days following surgery, could serve as evidence for precisely the opposite interpretation, an uncovering of innate preexisting connections between these senses (Chen et al., 2016; Bola et al., 2017; Maimon and Hemmo, 2022) or a re-calibration (Gallagher, 2020). This interpretation, which we believe is warranted by the findings, supports an amodal understanding of brain development and structure. This is further supported by the results of this study concerning spatial grouping based on geometric cues. This interpretation is supported by prior research conducted in our lab that has shown that the lateral-occipital tactile-visual area (LOtv) is an area activated by visual and tactile exploration of objects (Amedi et al., 2002) that can also be activated in the blind for processing object shapes after training with a visual to auditory sensory substitution device, indicating that this area is involved in the task of processing the geometry and shape of objects, irrespective of the sensory modality through which the information was conveyed (Amedi et al., 2007).

### 4.4. Replication and expansion of previous studies on the spontaneous emergence of geometry concepts in congenital cataracts

Spatial grouping tasks based on geometric cues have been used in prior research to show that spontaneous geometry intuitions arise independently from education in normally sighted Amazonian adults (Dehaene et al., 2006). This research was later expanded in our lab, showing that geometric knowledge and reasoning develop irrespective of vision (Heimler et al., 2021). This study showed that both normally sighted blindfolded people and the congenitally blind showed geometrical sense driven by touch alone. The results of this current study further strengthen and elaborate on these findings, now repeating the task for the first time *via* vision. The four children tested on this task in our study had very little formal education, with RS having never attended school at all and the other three children reaching up to second-grade education. The findings showed that all four children (RS and HB, who underwent surgery for bilateral congenital cataracts, and AC and GH, who underwent surgery for unilateral trauma-induced cataracts) performed above chance level, with RS and HB performing better than their peers who were born with intact vision. These findings further support the amodal nature of the brain, at least for geometry, and the innate preexisting links between the senses. More generally, these findings support the revised “neuronal recycling theory (Dehaene, 2005; Dehaene and Cohen, 2007)” that posits a task-selective, sensory independent interpretation of specialization in the brain (Striem-Amit et al. 2011, 2012a; Reich et al., 2012; Heimler et al., 2015; Amedi et al., 2017). Under this interpretation, areas such as the visual cortex are not “visual” *per se* as they do not undergo specialization for vision but rather undergo specialization for performing a specific task (where usually vision is the most accurate and easy way to perform the task) and thereby can be activated by corresponding information delivered through other senses. For example, the Lateral Occipital Cortex (LOC), commonly correlated with visual object and shape recognition, could be recruited for processing 3D geometric shapes, irrespective of the sensory modality through which the information was provided, as was indeed shown in Amedi et al. (2001).

Another example would be the Visual Word Form Area (VWFA) commonly associated with visual letter recognition. According to the task selective, sensory independent interpretation, this area would be designated for the task of symbol-to-phoneme conversion (independent of the visual modality), as supported by Striem-Amit et al. (2012b). There are many more such examples of task selectivity as opposed to sensory-dependent organization. For a full review, see Amedi et al. (2017). Similar views of the brain as a-modal or supramodal (see Pascual-Leone and Hamilton, 2001; Kupers and Ptito, 2011; Ricciardi et al., 2014; Cecchetti et al., 2016) fit well with this notion and theory.

### 4.5. Limitations and future directions

A practical limitation of the study pertains to the partial nature of the children’s medical histories, which were reported by the parents, and the lack of digitized medical data related to the children and their medical reports (the post-operative surgical report was handwritten). Due to this partial or illegible information, we did not, for example, have data concerning some of the children’s pre-op visual acuity. Furthermore, as these results represent the individual cases of the experiences of a number of children, the results cannot be generalized. Yet, we feel that this research indicates several future study directions. With respect to future directions, we suggest implementing this battery of tests on children undergoing congenital cataract removal, with data acquired pre-surgery, immediately following surgery, and months after surgery. This way, the progressive development can be tracked, further shedding light on the questions and issues we have discussed. In addition, future research on the neural underpinnings of children’s visual recovery in similar circumstances is warranted to further elucidate the link between the behavioral and the neurological. As such, fMRI studies can be conducted pre and post-surgery to investigate the mechanisms in the brain corresponding to the visual experiences of the children following surgery. This study presents a select few of the many lessons to be learned from these cases regarding the deepest aspects of visual development specifically and the profound interaction between the sensory experience and the brain more generally.

## 5. Conclusion

This study focused on the visual and geometry abilities of children who had undergone cataract removal surgery at Quiha hospital in Ethiopia as part of the Himalayan Cataract Project. The findings of the study reveal, first and foremost, that out of the cohort of children, those with congenital cataracts did not exhibit binocular rivalry, nor did they show depth perception when tested with the Brock string test. These two tests clearly delineated the congenitally blind children from the normally sighted at birth (who developed cataracts later in life). As such, we novelly propose the utilization of these two tests in retroactively confirming the blindness status of a child, particularly in cases where medical history and records are lacking. In addition, the current study replicates and expands upon previous studies conducted on cross-modal correspondence following congenital cataract removal in children. The children in this study reached nearly ceiling-level performance on the cross-modal correspondence task when tested a mere few days following their surgeries. Finally, this study strengthens the findings of previous studies indicating that geometry concepts arise independently from experience and education, thus supporting a task-selective, sensory-independent interpretation of specialization and development in the brain.

## Data availability statement

The original contributions presented in this study are included in the article/supplementary material, further inquiries can be directed to the corresponding author.

## Ethics statement

Ethical approval was not provided for this study on human participants because the study was conducted while the children were under the care and supervision of the local Quiha Hospital medical staff, with the parents present. The parents of all of the children signed written consent forms approving the participation of the children in the study. Written informed consent to participate in this study was provided by the participants’ legal guardian/next of kin. Written informed consent was obtained from the participant’s next of kind for the publication of any identifiable images or data included in this article.

## Author contributions

AM: writing—original draft, review, and editing, conceptualization, visualization, and project administration. ON and BH: conceptualization, investigation, and visualization. AA: writing—original draft, review, and editing, project administration, supervision, resources, conceptualization, investigation, methodology, and funding acquisition. All authors contributed to the article and approved the submitted version.
